# Leishmanicidal Activity of Nine Novel Flavonoids from *Delphinium staphisagria*

**DOI:** 10.1100/2012/203646

**Published:** 2012-05-22

**Authors:** Inmaculada Ramírez-Macías, Clotilde Marín, Jesús G. Díaz, María José Rosales, Ramón Gutiérrez-Sánchez, Manuel Sánchez-Moreno

**Affiliations:** ^1^Department of Parasitology, University of Granada, Severo Ochoa s/n, 18071 Granada, Spain; ^2^Departmento de Química Orgánica y Instituto Universitario de Bio-orgánica “Antonio González”, Universidad de La Laguna, 38206 La Laguna, Spain; ^3^Department of Statistics, University of Granada, Severo Ochoa s/n, 18071 Granada, Spain

## Abstract

*Objectives*. To evaluate the *in vitro* leishmanicidal activity of nine flavonoid derivatives from *Delphinium staphisagria* against *L. infantum* and *L. braziliensis*. *Design and Methods*. The *in vitro* activity of compounds **1**–**9** was assayed on extracellular promastigote and axenic amastigote forms and on intracellular amastigote forms of the parasites. Infectivity and cytotoxicity tests were carried on J774.2 macrophage cells using Glucantime as the reference drug. The mechanisms of action were analysed performing metabolite excretion and transmission electronic microscope ultrastructural alteration studies. *Results*. Nine flavonoids showed leishmanicidal activity against promastigote as well as amastigote forms of *Leishmania infantum* and *L. braziliensis*. These compounds were nontoxic to mammalian cells and were effective at similar concentrations up to or lower than that of the reference drug (Glucantime). The results showed that 2^″^-acetylpetiolaroside (compound **8**) was clearly the most active. *Conclusion*. This study has demonstrated that flavonoid derivatives are active against *L. infantum* and *L. braziliensis*.

## 1. Introduction

Leishmaniasis is an infection caused by different species of the protozoan genus *Leishmania*, which is transmitted by dipterans of the genera *Phlebotomus* in the Old World and *Lutzomyia* in the New World.

Leishmaniasis represents one of the most significant of a collection of neglected tropical diseases. According to the latest report of the World Health Organization [1], 350 million people in 88 countries are considered at risk of contracting leishmaniasis, and some 2 million new cases occur annually.

Drug treatment for leishmaniasis has been available since the beginning of the 20th Century [1], but only a few drugs have been developed for use and there are numerous drawbacks to each of the treatments. Two pentavalent antimonials are available (meglumine antimoniate or Glucantime and sodium stibogluconate or pentostan), but they have common side effects such as anorexia, vomiting, nausea, abdominal pain, malaise, and myalgia. They are available in several formulations, which are administered by intravenous infusion, including liposomal amphotericin B, amphotericin B lipid complex, and amphotericin B colloidal dispersion. However, side effects such as fever, chills, rigor and back pain, and transient nephrotoxicity or thrombocytopenia occur in some patients. Other antileishmanial medicines are paromomycin (aminosidine) and pentamidine isethionate, which are usually administered intramuscularly. The alkyl phospholipid (hexadecylphosphocholine), commonly known as miltefosine, induces gastrointestinal side effects such as anorexia, nausea, vomiting (38%) and diarrhea (20%), and also skin allergies, elevated hepatic transaminase concentrations, and, occasionally, renal failure. It is now used in combination with different classes of azole oral antifungal agents including ketoconazole, fluconazole, and itraconazole.

In addition to the adverse effects of the drugs, resistance to these treatments is appearing in the parasites. For all these reasons, further novel drug development is necessary to treat these infections.

Considerable attention is currently being paid to phytotherapy in the search for new drugs. One method used to discover new drugs is to investigate natural products from plants used medicinally [2]. The broad range of plant families and species available offers many potentially active leishmanicidal substances [3, 4]. Leishmanicidal capacity of alkaloid compounds isolated from medicinal plants has been studied [5], and the antileishmania capacity of Aloe Vera exudate against *L. donovani *has been established [6].

Leishmanicidal properties may reside in phytochemicals such as flavonoids, which are hence strong candidates for use in combination therapy against these infections. Flavonoids are abundant in fruits, vegetables, and saps of plants and are demonstrated to have anticarcinogenic, antimicrobial, and antiparasitic activity [7, 8]. Flavonoid analogues derived from *Consolida oliveriana *exerted a significant effect on the *in vitro* growth of species of *Leishmania *spp. [9].

In this work, the inhibitory effects of flavonoids from aerial parts of *Delphinium staphisagria* L. (Ranunculaceae) on the extracellular and intracellular stages of *L. infantum *and *L. braziliensis* were investigated. In addition, the cytotoxic effects of these compounds against a host cell line were assessed. We also used ^1^H-NMR spectroscopic analysis to determine the nature and percentage of the excretion metabolites and to elucidate any inhibitory effect that the compounds have on the glycolytic pathway. Finally, the effects of the compounds on the ultrastructure were studied.

## 2. Material and Methods

### 2.1. Plant Material

**Figure 1:**
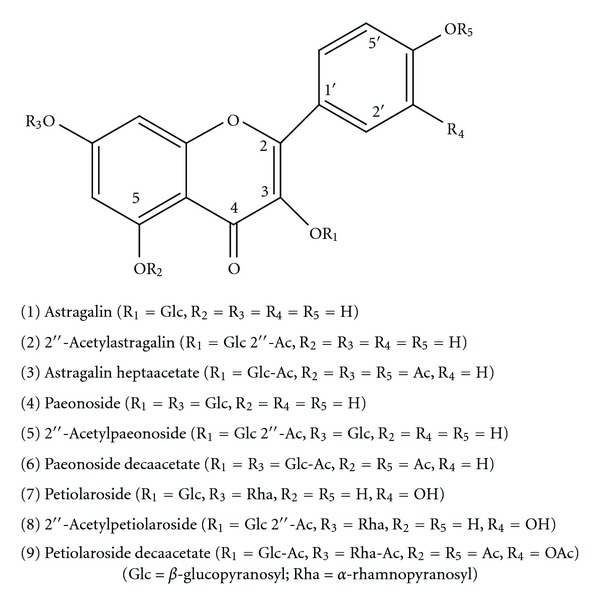
Flavonoid compounds investigated.

Aerial parts of the *Delphinium staphisagria* were collected and processed as described previously Díaz et al. [10]. Nine flavonoids (**1**–**9**) were isolated, derivatized, and identified (Figure 1) [10].

### 2.2. Parasite Strain and Culture

*L. infantum* (MCAN/ES/2001/UCM-10) and *L. braziliensis* (MHOM/BR/1975/M2904) were cultivated *in vitro* in trypanosomes liquid medium (MTL) with 10% inactive fetal bovine serum and were kept in an air atmosphere at 28°C, in Roux flasks (Corning, USA) with a surface area of 75 cm^2^, according to the methodology described by González et al. [4].

### 2.3. Cell Culture and Cytotoxicity Tests

J774.2 macrophages (ECACC number 91051511) were originally obtained from a tumour in a female BALB/c rat in 1968. The cytotoxicity test for macrophages was performed according to the methodology of González et al. [4]. After 72 hours of treatment, cell viability was determined by flow cytometry. Thus, 100 *μ*L/well of propidium iodide solution (100 mg/mL) was added and incubated for 10 min at 28°C in darkness. Afterwards, 100 *μ*L/well of fluorescein diacetate (100 ng/mL) was added and incubated under the same conditions. Finally, the cells were recovered by centrifugation at 400 g for 10 min and the precipitate washed with phosphate buffered saline (PBS). Flow cytometric analysis was performed with a FACSVantage flow cytometer (Becton Dickinson). The percentage viability was calculated in comparison with the control culture. The IC_50_ was calculated using linear regression analysis from the Kc values of the concentrations employed.

### 2.4. *In Vitro* Activity Assay

#### 2.4.1. Promastigote Forms, Assay

The compounds obtained were dissolved in the culture medium, at dosages of 100, 50, 25, 10, and 1 *μ*M. The effects of each compound against promastigote forms were tested at 72 hours using a Neubauer haemocytometric chamber. The antileishmanial effect is expressed as the IC_50_, that is, the concentration required to give 50% inhibition, calculated by linear regression analysis from the Kc values of the concentrations employed.

#### 2.4.2. Amastigote Forms, Assay

J774.2 macrophages were grown in minimum essential medium (MEM) plus glutamine (2 mM), supplemented with 20% inactive fetal bovine serum, and were kept in a humidified atmosphere of 95% air and 5% CO_2_ at 37°C.

Cells were seeded at a density of 1 × 10^4^ cells/well in 24-well microplates (Nunc) with rounded coverslips on the bottom and cultured for 2 days. Afterwards the cells were infected *in vitro* with promastigote forms of *L. infantum* and *L. braziliensis*, at a ratio of 10 : 1 during 24 hours. The non-phagocytosed parasites were removed by washing, and then the drugs (at 1, 10, 25, 50, 100 *μ*M) were added. Macrophages with the drugs were incubated for 72 hours at 37°C in 5% CO_2_.

Drug activity was determined on the basis of number of amastigotes in treated and untreated cultures in methanol-fixed and Giemsa-stained preparations. The number of amastigotes was determined by analyzing 200 host cells distributed in randomly chosen microscopic fields. The antileishmanial effect is expressed as the IC_50_ values are the means of three separate determinations.

#### 2.4.3. Axenic Amastigote Forms, Assay

Axenic amastigotes forms of *L. infantum* and *L. braziliensis *were cultured following the methodology described previously by Moreno et al. [11]. Thus, promastigote transformation to amastigotes was obtained after three days of culture in M199 medium (Invitrogen, Leiden, The Netherlands) supplemented with 10% heat-inactivated FCS, 1 g/L *β*-alanine, 100 mg/L L-asparagine, 200 mg/L sacarose, 50 mg/L sodium pyruvate, 320 mg/L malic acid, 40 mg/L fumaric acid, 70 mg/L succinic acid, 200 mg/L *α*-ketoglutaric acid, 300 mg/L citric acid, 1.1 g/L sodium bicarbonate, 5 g/L MES, 0.4 mg/L hemin, and 10 mg/L gentamicin pH 5.4 at 37°C. The effect of each compound against axenic amastigotes forms was tested at 48 hours using a Neubauer haemocytometric chamber. The antileishmanial effect is expressed as the IC_50_.

### 2.5. Infection Assay

J774.2 macrophage cells were grown under the same conditions expressed in amastigote forms assay during two days. Afterwards, the cells were infected *in vitro *with promastigote forms of *L. infantum *and *L. braziliensis*, at a ratio of 10 : 1. The drugs (IC_25_ concentrations) were added immediately after infection and were incubated for 12 hours at 37°C in 5% CO_2_. The nonphagocytosed parasites and the drugs were removed by washing, and then the infected cultures were grown for 10 days in fresh medium. Fresh culture medium was added every 48 h. The drug activity was determined from the percentage of infected cells and the number of amastigotes per infected cell (in treated and untreated cultures) in methanol-fixed and Giemsa-stained preparations. The percentage of infected cells and the mean number of amastigotes per infected cell were determined by analyzing 200 host cells distributed in randomly chosen microscopic fields. Values are the average of three separate determinations.

### 2.6. Metabolite Excretion

Cultures of *L. infantum* and *L. braziliensis* promastigotes (initial concentration 5 × 10^5^ cells/mL) received IC_25_ concentrations of the compounds (except for control cultures). After incubation for 96 hours at 28°C, the cells were centrifuged at 400 g for 10 min. Then supernatants were collected to determine the excreted metabolites using ^1^H-NMR, and, eventually, chemical displacements were expressed in parts per million (ppm), using sodium 2,2-dimethyl-2-silapentane-5-sulphonate as the reference signal. The chemical displacements used to identify the respective metabolites were consistent with those described by Fernández-Becerra et al. [12]

### 2.7. Ultrastructural Alterations

The parasites were cultured at a density of 5 × 10^5^ cells/mL in MTL medium, and the cultures contained drugs at the IC_25_ concentration.

After 96 hours, those cultures were centrifuged at 400 g for 10 min, and the pellets produced were washed in PBS and then mixed with 2% (v/v) P-formaldehyde-glutaraldehyde in 0.05 M cacodylate buffer (pH 7.4) for 4 hours at 4°C. After that, the pellets were prepared for TEM employing the technique of González et al. [4]. 

## 3. Results

### 3.1. *In Vitro* Antileishmanial Evaluation

The IC_50_ values obtained on promastigote, axenic amastigote, and intracellular amastigote forms of *L. infantum* and *L. braziliensis* after 72 h of exposure with compounds **1**–**9** are displayed in the first three columns of Table 1. Values of the reference drug, Glucantime, are also included for all cases for comparison.

The antileishmanial activity in both extra- and intracellular forms is similar or, in most cases, less than that found for Glucantime, with the compounds **7**, **8**, and **9** presenting the lowest IC_50_.

The macrophage toxicity of these compounds is of particular interest as all compounds tested show significantly less toxicity to macrophages than the reference drug, from 15 to 65 times (Table 1). Thus, compounds **4**,** 5**,** 6**,** 7**, and** 8 **present IC_50_ values greater than 1000 *μ*M, while compounds **1**,** 2**,** 3**, and** 9** give smaller IC_50_ values of 235.9 *μ*M, 267.0 *μ*M, 350.6 *μ*M, and 489.8 *μ*M, respectively. 

Toxicity values substantially influence the more informative selectivity index data (SI, IC_50_ macrophage toxicity/IC_50_ activity of extracellular or intracellular forms of the parasite) which are shown in the last three columns of Table 1. The numbers of times that the compound SI exceeds the Glucantime SI are given in brackets. These values are very illustrative of the *in vitro* potential of the compounds tested with respect to the reference drug. Compounds **7**, **8**, **4**, **5**, and **6** showed the best SI for *L. infantum *(Table 1(a)), with indexes exceeding that of the reference drug SI by more than 50 times. Compounds **1**, **2**, **3**, and **9** were more effective than Glucantime; however, SI values of 50 times or greater were not reached, which is the criterion for a compound to be included in subsequent studies [13].

Similar results can be extracted from the *L. braziliensis* data shown in Table 1(b). The compounds **7**, **8**, **4**, and **6** again gave the best SI results in the three assays performed, with values exceeding those of the reference drugs by 79, 103, and 59 times in the case of compound **7**, by 58, 78, and 100 times for compound **8**, by 59, 84, and 61 times for compound **4**, and by 53, 71, and 66 times for compound **6. **Although compound **5** did not give SI values greater than or equal to 50 times those of the reference drugs, it was included in subsequent studies because promastigote and intracellular amastigote SI values were close to 50 times that of the reference drug, while the axenic amastigote SI was two times (Table 1(b)).

**Figure 2:**
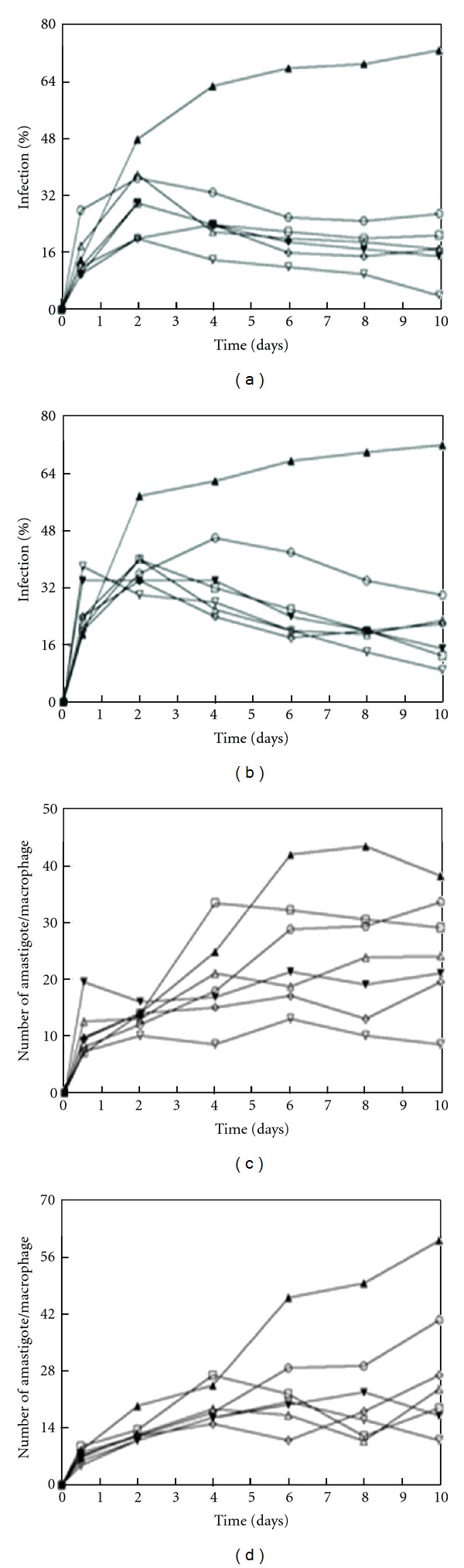
Effects of flavonoids **4**,** 5**,** 6**,** 7**, and** 8 **on the infection and growth rates of *Leishmania *spp. (a) rate of infection of *L. infantum*; (c) mean number of amastigotes per infected J774 A.2 macrophage cells of *L. infantum*; (b) rate of infection of *L. braziliensis*; (d) mean number of amastigotes per infected J774 A.2 macrophage cells of *L. braziliensis*. (-▲-, control; -∆-, comp. **4**; -*▼*-, comp.** 5**; -□-, comp. **6**; -*◊*-, comp. **7**; -*∇*-, comp. **8**; -○-, Glucantime). At IC_25_ conc., values are means of three separate experiments.

The effectiveness of the compounds on the infection rate and the intracellular replication of the amastigote forms was determined by the infection assay (Figure 2). When selected flavonoid compounds **4**–**8** and Glucantime were added at their respective IC_25_ concentrations to macrophages infected with *Leishmania* spp. promastigote forms, the infection rate decreased significantly after 12 h with respect to the control measurement. The infection rate decreases followed the trend **8 **> **5 **> **7 **= **4 **> **6** for *L. infantum *(Figure 2(a)) and **8** > **6** > **5** > **7** > **4** for *L. braziliensis* (Figure 2(b)) with percentages of inhibition capacity of 95%, 79%, 77%, and 71%, respectively, in the case of *L. infantum* and 88%, 82%, 79%, 69%, and 68%, respectively, in the case of *L. braziliensis. *These values are remarkably higher than those for inhibition by Glucantime (63% and 58% for *L. infantum* and *L. braziliensis,* resp.).

All five compounds were more effective than Glucantime (only 12% decrease for *L. infantum* and 33% decrease for *L. braziliensis*) at decreasing the average number of amastigotes per infected macrophage cells (Figures 2(c) and 2(d)). Compound **8** was the most effective in *L. infantum *and *L. braziliensis. *The amastigote number decreases measured were as follows: **8** (78%) > **7** (49%) > **5 **(45%) > **4** (37%) > **6** (24%) for *L. infantum *and **8** (82%) > **5** (72%) > **6** (69%) > **4** (61%) > **7** (55%) for *L. braziliensis.*

### 3.2. Metabolites Excretion Effect

**Figure 3:**
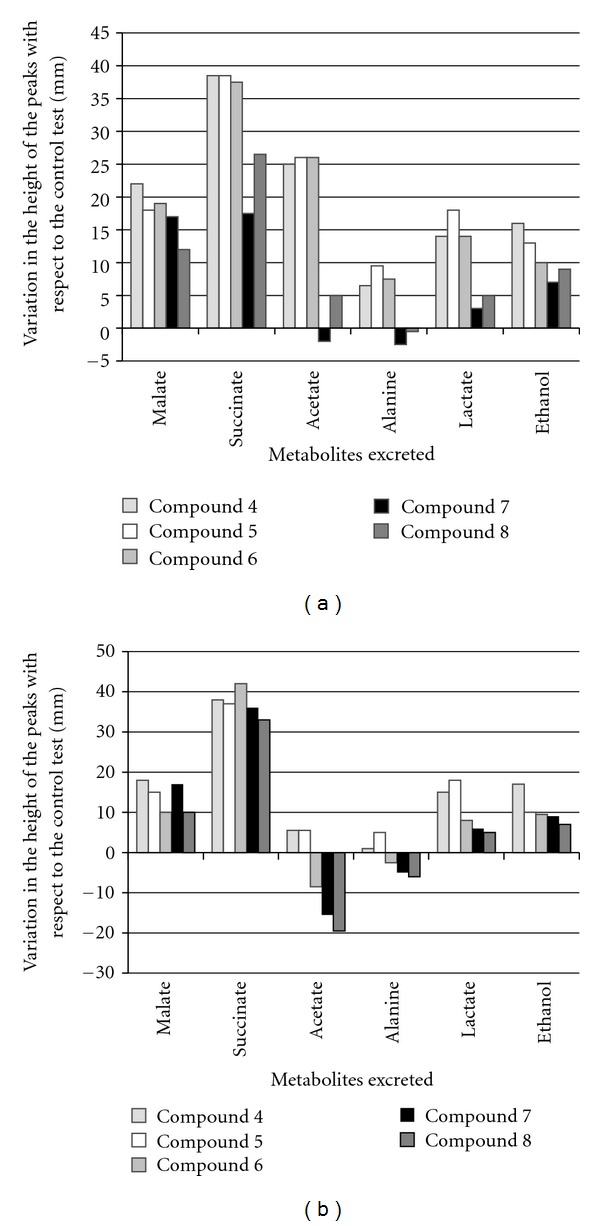
Variation in the height of the peaks corresponding to catabolites excreted by *L. infantum* (a) and *L. braziliensis* (b) promastigote forms in the presence of flavonoid derivatives with respect to the control test.

After treatment of the parasites with compounds **4**–**8** at IC_25_, the excretion of the catabolites was clearly altered.  Figure 3 displays the modifications observed in the height of the spectra peaks corresponding to the most representative final excretion products. From a careful examination of the data, it would appear that there are marked differences in the catabolic pathway. In the case of *L. infantum*, compounds **4**,** 5**,** 6**, and **8** gave similar spectra, with an increase in the excretion of succinate, and acetate, followed by malate, alanine, lactate and ethanol. On the other hand, compound **7** causes an increase in the production of malate, succinate, ethanol, and lactate, but a decrease of excreted acetate and alanine levels (Figure 3(a)).

In the case of *L. braziliensis, *the increase of excreted succinate was the most significant change when the parasites were treated with the compounds. Compounds **6**,** 7**, and** 8** caused decreases in the excretion of acetate (Figure 3(b)).

### 3.3. Ultrastructural Alterations

**Figure 4:**
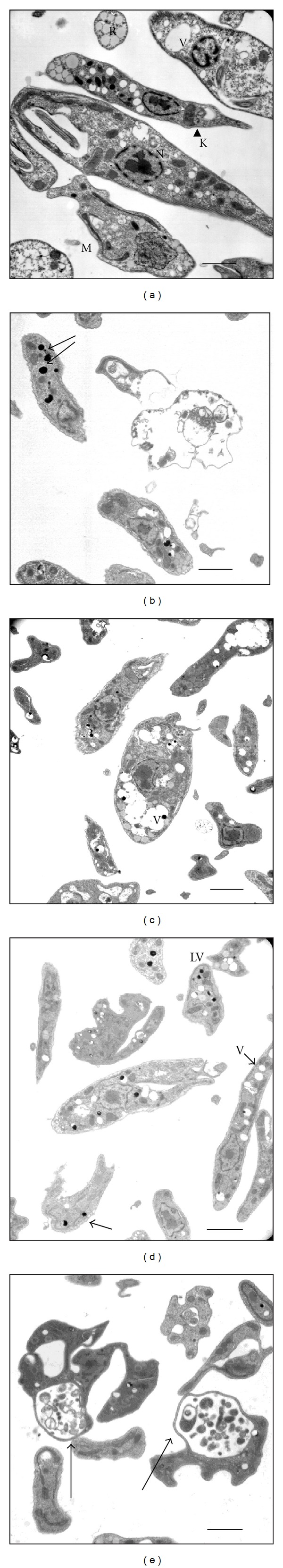
Ultrastructural alterations by TEM in *L. infantum* treated with the flavonoid derivatives. (a) Control parasite of *L. infantum* showing organelles with their characteristic features such as the nucleus (N), kinetoplast (K), reservosomes (R), mitochondrion (M), and vacuoles (V) (bar: 1 *μ*m). (b) *L. infantum *treated with compound **4**. Electrodense organelles (arrow) (Bar: 1 *μ*m). (c) *L. infantum* treated with compound **5** with intense vacuolization (V) (Bar: 1.59 *μ*m). (d) *L. infantum* treated with compound **6**, vacuoles (V), lipid vacuoles (LV), and electrodense vesicles (arrow) (bar: 1.59 *μ*m). (e) *L. infantum* treated with compound **8** with distorted parasites (arrow) (Bar: 1 *μ*m).

**Figure 5:**
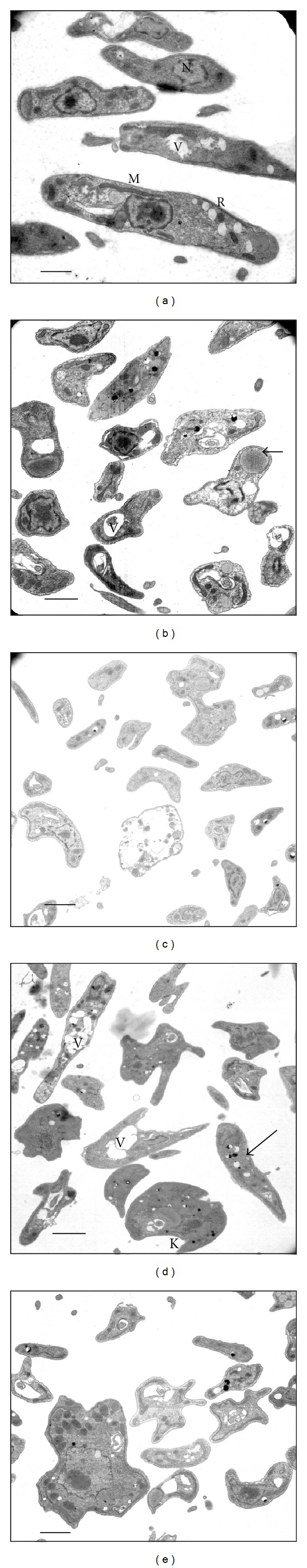
Ultrastructural alterations by TEM in *L. braziliensis* treated with flavonoid derivatives. (a) Control parasite of *L. braziliensis* with structures such as the nucleus (N), reservosomes (R), vacuoles (V), and mitochondrion (M) (bar: 1 *μ*m). (b) *L. braziliensis* treated with compound **4** with vacuoles (V) and swelling kinetoplast (arrow) (bar: 1 *μ*m). (c) *L. braziliensis* treated with compound **6** (bar: 1.59 *μ*m). (d) *L. braziliensis* treated with compound **7** with intense vacuolization (V), electrodense organelles (arrow), and swelling kinetoplast (K) (bar: 2.33 *μ*m). (e) *L. braziliensis* treated with compound **8** with distorted forms (bar: 1.59 *μ*m).

The transmission electron microscope evaluation of flavonoid compounds **4**–**8** against *Leishmania* spp. promastigotes showed notable ultrastructural alterations, as reflected in Figures 4 and 5 (panels b, c, d, e) with respect to the control (Figures 4(a) and 5(a)). All compounds produced significant alterations to *L. infantum*, but the compounds most effective were **4** and **8**, as illustrated in Figures 4(b) and 4(e). All of the compounds induced the formation of strongly electrodense inclusions that appeared inside and outside vacuoles with different sizes, or directly in the cell cytoplasm. The major effect of compound **8** was that many of the parasites adopted distorted shapes with distorted cytoplasm, while no cytoplasmic organelles were visible due to the altered appearance of promastigotes. However, large vacuoles containing cellular debris were visible. With compound **4 **(Figure 4(b)), many of the parasites appeared dead, while another showed pronounced vacuolization, and many of these vacuoles contained strongly electrodense inclusions. Compound **5** produced similar effects (Figure 4(c)). In the same way, compound **6** induced empty vacuoles, lipid vacuoles, and a large number of electrodense vesicles (Figure 4(d)). By contrast, compound **7 **showed no induction of substantial alterations (graph not shown).

Studies of ultrastructural alterations made on *L. braziliensis *showed that the compounds produced changes similar to those they produced in *L. infantum. *Another effect of the compounds was the expansion of the kinetoplast, which appeared swollen when treated with compound **4** (Figure 5(b)). Compound **8** (Figure 5(e)) induced changes in the shape of parasites, with them showing completely altered forms. All these changes occurred after treatment with compound **7** (Figure 5(d)), while compound **5** produced no significant changes compared to the control (data not shown).

## 4. Discussion

Many people living in rural areas have no easy access to conventional allopathic treatments, largely due to the limited availability of health services and their low socioeconomic status. Therefore, plants may provide an important and necessary source of therapeutic medicinal compounds. Flavones and their derivatives, flavonoids and flavonols, are among the most attractive plant derivatives that might enrich the current therapy options, due to their extremely large range of biological properties [14].

In this study, *in vitro* biological activity of nine flavonoid compounds against extracellular and intracellular forms* Leishmania* spp. was investigated. This is important as many studies of the activity of compounds against *Leishmania* spp. are performed on promastigote forms, which are much easier to work *in vitro.* However, since extracellular forms are not the developed forms of the parasite in vertebrate hosts, evaluations made with these forms are merely indicative of the potential leishmanicidal activity of the compounds tested. Consequently, a preliminary test using extracellular promastigote forms should always be complemented by a subsequent evaluation using intracellular forms (amastigotes in vertebrate host cells), so that a better understanding of the activity may be obtained [4].

The petiolaroside-based derivatives (**7–9**) presented the lowest IC_50 _values against extra- and intracellular forms.

Previously, the nine flavonoids investigated here were tested against epimastigote forms of *Trypanosoma cruzi*, and the most effective compounds were astragalin (**1**) and two acetylated derivatives (**2** and** 3**). These had significantly lower IC_50_ values compared with benznidazole [15]. By contrast, in this study, we saw that the astragalin and its derivates were the least effective agents against *Leishmania* spp.

Petiolaroside, 2′′-acetylpetiolaroside (**7** and **8**, resp.), and paeonoside and its derivates (**4**,** 5, **and** 6**) showed the best selectivity indexes. These indexes exceeded the reference drug SI by more than 50 times. The astragalin and its derivates (**1**,** 2, **and** 3**) and petiolaroside decacetate (**9**) were all more effective than Glucantime.

If we compare the results obtained for the infection rate and the number of amastigotes per infected macrophage cells for both *Leishmania* species, it can be concluded that 2′′-acetylpetiolaroside (**8**) is clearly the most active. The acetylated derivatives 2′′-acetylpetiolaroside (**8**) and 2′′-acetylpaeonoside (**5**) are more active than petiolaroside (**7**) and paeonoside (**4**), respectively.

As far as is known to date, none of the trypanosomatids studied are capable of completely degrading glucose to CO_2_ under aerobic conditions. When this occurs, it results in the excretion of a major part of the carbon skeleton into the medium as fermented metabolites, which can differ according to the employed species [16]. ^1^H-NMR spectra enabled us to determine the fermented metabolites excreted by the parasites during their *in vitro* culture. The final products of glucose catabolism in *Leishmania* are usually CO_2_, succinate, acetate, L-lactate, pyruvate, L-alanine, and ethanol [17]. As with *T. cruzi* [18], one of the major metabolites excreted by *Leishmania* spp. is succinate, with the main role likely to be to maintain the glycosomal redox balance by providing two glycosomal oxidoreductase enzymes. These enzymes allow reoxidation of NADH produced by glyceraldehyde-3-phosphate dehydrogenase in the glycolytic pathway. Succinic fermentation offers one significant advantage as it requires only half the produced phosphoenolpyruvate (PEP) to maintain the NAD+/NADH balance. The remaining PEP is converted to acetate, depending on the species under consideration.

The majority of metabolites excreted by* L. infantum* are succinate and acetate. In the case of *L. infantum, *variations in the final catabolism products seem to be dependent on the structural aspects of the compounds assayed. These data agree well with those of other authors [18].

Analyses of *in vitro* and *in vivo* results of *Trypanosoma cruzi *showed that the best potential flavonoids to treat Chagas disease were the acetylated compounds **2**, **5**, and **6 **[15]. The present work confirmed this conclusion in the case of visceral leishmaniasis (*L. infantum*) and mucocutaneous leishmaniasis (*L. braziliensis*). Moreover, these data are consistent with others previously published, which showed that several acetylated flavonoids derived from *Consolida oliveriana *were very active *in vitro *against both extracellular and intracellular forms of *L. (V.) peru*v*iana *and *L. (V.) braziliensis *[9].

In conclusion, this study has demonstrated that flavonoid derivatives are active against *L. infantum *and *L. braziliensis.* These results support further investigation of flavonoid compounds as potential agents against Leishmaniasis.

## Figures and Tables

**Table 1a:** (a) Leishmania infantum

Compounds	Activity IC_50_ (*μ*M)^a^			^ b^Macrophage toxicity IC_50_	SI^c^		
	Promastigote forms	Axenic amastigote forms	Intracellular amastigote forms		Promastigote forms	Axenic amastigote forms	Intracellular amastigote forms
Glucantime	18.0 ± 3.1	24.2 ± 2.6	30.0 ± 2.7	15.2 ± 1.3	0.8	0.6	0.5
**1**	35.1 ± 3.3	29.2 ± 2.8	45.9 ± 2.7	235.9 ± 17.9	6.7 (8)	8.1 (14)	5.1 (10)
**2**	34.1 ± 1.6	32.7 ± 5.0	42.3 ± 3.9	267.0 ± 11.0	7.8 (10)	8.2 (14)	6.3 (13)
**3**	40.4 ± 3.4	29.2 ± 3.1	38.1 ± 1.6	350.6 ± 26.8	8.7 (11)	12.0 (20)	9.2 (18)
**4**	25.1 ± 1.9	26.9 ± 1.4	31.4 ± 2.0	>1000.0 ± 60.0	39.8 (50)	37.2 (62)	31.8 (64)
**5**	24.6 ± 3.7	29.8 ± 2.2	37.2 ± 3.5	>10000 ± 71.4	40.7 (51)	33.6 (56)	26.9 (54)
**6**	23.1 ± 4.6	30.5 ± 2.3	40.3 ± 2,4	>1000.0 ± 82.0	43.3 (54)	32.8 (55)	24.8 (50)
**7**	24.5 ± 1.1	19.4 ± 1.4	27.8 ± 1.4	>1000.0 ± 55.6	40.8 (51)	51.5 (86)	36.0 (72)
**8**	19.1 ± 0,9	18.4 ± 1.3	32.1 ± 2.0	>1000.0 ± 38.9	52.4 (66)	54.3 (91)	31.2 (62)
**9**	22.8 ± 1.7	21.5 ± 0.9	31.0 ± 1.7	489.8 ± 22.4	21.5 (27)	22.8 (38)	15.8 (32)

**Table 1b:** (b) Leishmania braziliensis

Compounds	Activity IC_50 _(*μ*M)^a^			^ b^Macrophage toxicity IC_50_	SI^c^		
	Promastigote forms	Axenic amastigote forms	Intracellular amastigote forms		Promastigote forms	Axenic amastigote forms	Intracellular amastigote forms
Glucantime	25.6 ± 1.6	30.4 ± 6.1	31.1 ± 3.0	15.2 ± 1.3	0.6	0.5	0,5
**1**	28.1 ± 1.0	22.6 ± 1.7	32.2 ± 1.6	235.9 ± 17.9	8.4 (14)	10.4 (21)	7.3 (15)
**2**	25.1 ± 2.5	27.9 ± 2.7	30.2 ± 3.8	267.0 ± 11.0	10.6 (18)	9.6 (19)	8.8 (18)
**3**	28.4 ± 3.0	32.9 ± 3.0	41.9 ± 4.0	350.6 ± 26.8	12.3 (21)	10.7 (21)	8.4 (17)
**4**	28.1 ± 5.2	23.7 ± 2.1	32.7 ± 2.1	>1000.0 ± 60.0	35.6 (59)	42.2 (84)	30.6 (61)
**5**	34.1 ± 6.6	19.4 ± 0.9	50.9 ± 6.2	>1000.0 ± 71.4	29.3 (49)	51.5 (103)	20.0 (40)
**6**	31.3 ± 3.0	28.0 ± 1.6	30.4 ± 2.7	>1000.0 ± 82.0	31.9 (53)	35.7 (71)	32.9 (66)
**7**	21.1 ± 1.7	19.5 ± 0.8	33.9 ± 3.5	>1000.0 ± 55.6	47.4 (79)	51.3 (103)	29.5 (59)
**8**	29.0 ± 1.5	25.5 ± 0.7	20.1 ± 1.7	>1000.0 ± 38.9	34.5 (58)	39.2 (78)	49.8 (100)
**9**	32.3 ± 3.3	24.6 ± 1.3	21.0 ± 1.3	489.8 ± 22.4	15.2 (25)	19.9 (40)	23.3 (47)

Results are averages of three separate determinations.

^a^IC_50_ = the concentration required to give 50% inhibition, calculated by linear regression analysis from the Kc values at concentrations employed (1, 10, 25, 50, and 100 *μ*M).

^b^Against J774.2 macrophages after 72 h of culture.

^c^Selectivity index = IC_50_ macrophages/IC_50_ extracellular and intracellular form of the parasite. In brackets: number of times that compound SI exceeds the reference drug SI.
